# Post-Treatment Response and Safety of Stem Cell-Based Urethral Injection Therapy for Female Stress Urinary Incontinence: A Systematic Review and Meta-Analysis

**DOI:** 10.3390/jcm15197737

**Published:** 2026-10-08

**Authors:** Shadha Nasser Mohammed Bahutair, Sana Fathima, Ketaki Devendra Tayade, Areebah Akhter, Rajani Dube, Mohamedanas Mohamedfaruk Patni, Rasha Aziz Attia Salama

**Affiliations:** 1 Department of Obstetrics and Gynecology, RAK College of Medical Sciences, Ras Al Khaimah Medical and Health Sciences University, Ras Al Khaimah P.O. Box 11172, United Arab Emirates; rajani.dube@rakmhsu.ac.ae; 2 RAK College of Medical Sciences, Ras Al Khaimah Medical and Health Sciences University, Ras Al Khaimah P.O. Box 11172, United Arab Emirates; sana.21901107@rakmhsu.ac.ae (S.F.); ketaki.21901052@rakmhsu.ac.ae (K.D.T.); areebah.21901072@rakmhsu.ac.ae (A.A.); 3 Department of Community and Family Medicine, RAK College of Medical Sciences, Ras Al Khaimah Medical and Health Sciences University, Ras Al Khaimah P.O. Box 11172, United Arab Emirates; mohamedanas@rakmhsu.ac.ae (M.M.P.); rasha.aziz@rakmhsu.ae (R.A.A.S.)

**Keywords:** stress urinary incontinence, stem cell therapy, cell-based therapy, regenerative medicine, urethral injection, post-treatment response, systematic review, meta-analysis

## Abstract

**Background/Objectives**: Cell-based therapies have been proposed as a regenerative treatment for female stress urinary incontinence (SUI), but their efficacy, safety, and optimal delivery characteristics remain uncertain. The aim of this review is to systematically evaluate the post-treatment clinical response and safety of cell-based injection therapies for female SUI and explore factors associated with post-treatment response. **Methods**: PubMed, Scopus, and the Cochrane Library were searched from inception to 15 February 2026, and the search was subsequently updated through 25 April 2026 to identify newly published studies. Randomized controlled trials and prospective single-arm studies evaluating urethral injection of cell-based therapies in adult women with SUI were included. Random-effects meta-analysis was used to pool study-defined post-treatment response proportions. Subgroup analyses examined response definition, cell source, urinary incontinence type, ultrasound guidance, injection approach, and post-treatment physiotherapy. Continuous moderators were explored using univariable meta-regression. Placebo-controlled randomized trials reporting a common 12-month response endpoint were additionally pooled using risk ratios (RR). **Results**: Twenty-one studies involving 885 women were included. Seventeen studies contributed to the pooled post-treatment response analysis. The overall response proportion was 63.7% (95% confidence interval [CI] 56.7–70.5%), with moderate-to-substantial heterogeneity (I^2^ = 62.6%). Exploratory subgroup analyses showed higher post-treatment response in ultrasound-guided cohorts than non-ultrasound-guided cohorts (78% vs. 57%; *p* < 0.001), in cohorts restricted to pure SUI compared with those including mixed urinary incontinence (76% vs. 55%; *p* < 0.001), and in cohorts incorporating post-treatment physiotherapy compared with those without physiotherapy (78% vs. 58%; *p* < 0.001). Two placebo-controlled randomized trials reporting ≥ 50% reduction in stress incontinence episode frequency at 12 months were included in an exploratory comparative meta-analysis. The analysis showed no significant difference between cell therapy and placebo (RR 1.02, 95% CI 0.85–1.23; *p* = 0.85). Reported adverse events were predominantly minor and transient, commonly comprising irritative urinary symptoms, urinary tract infection, and biopsy- or injection-site complications. Serious treatment-related adverse events were rare, and no treatment-related deaths were reported. **Conclusions**: Stem cell-based injection therapy for female SUI is associated with encouraging post-treatment response rates and a generally favorable short- to medium-term safety profile. However, this estimate cannot be interpreted as treatment-specific efficacy. The available placebo-controlled randomized evidence did not demonstrate a higher 12-month response with cell therapy than placebo. Cell-based therapy should therefore remain investigational, pending adequately powered, controlled trials and long-term follow-up.

## 1. Introduction

Stress urinary incontinence (SUI) is defined by the International Continence Society (ICS) as the involuntary leakage of urine on effort, exertion, coughing, or sneezing, and represents the most prevalent form of urinary incontinence globally [1,2]. Estimates consistently place the worldwide burden at over 200 million affected individuals, with the condition disproportionately impacting women [1,3,4]. Prevalence among adult women has been reported between 20% and 44% [5,6,7]. These figures are widely considered underestimates given the persistent social stigma that discourages patients from seeking medical evaluation [5,6,7]. The European Association of Urology (EAU)’s guidelines recognize SUI as a major quality-of-life condition requiring evidence-based management across both sexes [8].

Beyond its clinical impact, SUI places a growing economic burden on individuals and health systems. In the United Kingdom alone, direct annual expenditure on its management has historically exceeded £740 million [9]. More recently, the aggregate economic burden of urinary incontinence across the 27 European Union member states was estimated at €69.1 billion in 2023. The figure is projected to increase to €100.2 billion by 2030 if current trends continue without preventive intervention, with women bearing approximately four times the economic burden of men [10]. The condition imposes wide-ranging quality-of-life consequences including social withdrawal, psychological distress, sexual dysfunction, and functional limitation, further compounding its societal cost [6,11].

The pathophysiology of SUI is rooted in structural and neuromuscular compromise of the urethral sphincter complex and pelvic floor support [12]. The two primary mechanisms are urethral hypermobility arising from weakened bladder neck support, and intrinsic sphincter deficiency (ISD) which is characterized by failure of urethral closure, with most patients exhibiting a combination of both [13]. Progressive loss of rhabdosphincter muscle mass is a key contributor, with sphincter volume declining from approximately 88% at birth to around 34% by the ninth decade of life [4]. Histopathological studies of urethral tissue from women with urinary incontinence have demonstrated degeneration of the urethral sphincter and its supporting connective tissue [6]. These findings provide a biological rationale for investigating regenerative therapies for urinary incontinence.

The current management of SUI follows a stepwise approach. Conservative first-line strategies include pelvic floor muscle training, behavioral modification, bladder training, and vaginal devices or pessaries [11,14]. Pharmacological options, including duloxetine hydrochloride, have demonstrated limited efficacy and carry notable adverse effect profiles that have restricted regulatory approval in certain jurisdictions [15]. Urethral bulking agents offer a minimally invasive option for ISD but are associated with high recurrence rates and subjective failure approaching 90% in some series, requiring repeated procedures [14]. Surgical intervention has largely been anchored by the synthetic polypropylene mid-urethral sling (MUS), endorsed by both the Society of Urodynamics, Female Pelvic Medicine and Urogenital Reconstruction (SUFU) and the American Urogynecologic Society, and supported by the European Association of Urology (EAU) guidelines [8,16]. Despite its widespread clinical adoption, the mid-urethral sling introduces lifelong risk of synthetic mesh extrusion, urethral erosion, and complex, tissue-scarring mesh removal surgeries [17]. Reports of mesh-related reoperation reach 6.9% at nine years [18]. The existing treatments address symptoms without restoring the underlying anatomical and functional deficits which has encouraged exploration of options in regenerative medicine [19,20].

Stem cell therapy offers a theoretically curative approach to sphincter regeneration by virtue of the capacity of stems cells to differentiate into multiple cell lineages and exert paracrine effects on native tissue by secreting growth factors and cytokines [2,21,22]. Several cell types have been investigated for SUI. The most studied are muscle-derived stem cells (MDSCs), obtained from skeletal muscle biopsy [1,4]. Additional cell types under clinical investigations include adipose-derived stem cells (ADSCs), bone marrow-derived mesenchymal stem cells (BMSCs), and human umbilical cord blood stem cells [4,21,23]. Despite this growing body of evidence, cell-based therapy for SUI has not reached standardized clinical implementation.

This systematic review aims to synthesize the current clinical evidence on stem cell-based therapies for female SUI, estimate post-treatment response and safety, explore factors associated with treatment outcomes, and evaluate available comparative evidence from randomized controlled trials. Specifically, the review addresses the following questions: (1) What is the proportion of clinical response in women with SUI following stem cell-based injection therapy, and what is the associated safety profile? (2) What does the available randomized comparative evidence indicate regarding treatment-specific efficacy? (3) Which factors, such as cell types and injection site, may be associated with the post-treatment response? (4) What gaps in evidence remain that should inform the design of future randomized controlled trials?

## 2. Materials and Methods

This systematic review and meta-analysis were conducted and reported in accordance with the preferred reporting items for systematic reviews and meta-analyses (PRISMA) 2020 guideline [24]. A formal review protocol was not registered prospectively on PROSPERO or any equivalent register prior to the conduct of this review.

### 2.1. Eligibility Criteria

Studies were eligible for inclusion if they enrolled adult female patients with a clinical diagnosis of SUI and evaluated the efficacy and/or safety of a stem cell-based therapy administered by urethral injection. Both randomized controlled trials (RCTs) and single-arm prospective studies were eligible. No restriction was placed on minimum sample size or year of publication.

Regarding the measured outcome, no restriction was placed on outcome type; however, included studies were required to report at least one pre-specified, measurable treatment outcome with a clearly defined study-specific criterion for success to be eligible for inclusion in the meta-analysis. Accepted outcome domains included objective continence assessed by pad test, patient-reported incontinence severity using validated instruments such as the International Consultation on Incontinence Questionnaire—Urinary Incontinence Short Form (ICIQ-UI SF), Incontinence Quality-of-Life Questionnaire (I-QOL), Urogenital Distress Inventory-6 (UDI-6), Incontinence Impact Questionnaire-7 (IIQ-7) or equivalent quality-of-life measures, incontinence episode frequency, patient global impression of improvement, and adverse events.

Studies were excluded if they were conducted in animal models or reported preclinical data; enrolled exclusively male patients or mixed-sex cohorts from which female-specific outcomes could not be isolated; were published in a non-peer-reviewed format such as conference abstracts, editorials, letters; narrative reviews without original patient level data; or were not published in English language.

### 2.2. Search Strategy and Information Sources

A systematic search of PubMed, Scopus, and Cochrane Library electronic databases was conducted. Medical Subject Headings (MeSH) and free-text term keywords like “Urinary Incontinence, Stress”[Mesh] and “stress urinary incontinence” were used in combination “Stem Cells”[Mesh], “stem cell” to include studies between database inception to 15 February 2026. The searches were subsequently updated through 25 April 2026 to identify newly published eligible studies. To minimize the risk of missing eligible studies, the reference lists of included studies and relevant review articles identified during screening were hand-searched. No clinical trial registers or grey literature sources were searched.

All records retrieved from the three databases were imported into a shared reference management system and deduplicated. Three reviewers independently screened all titles and abstracts against the pre-defined eligibility criteria, followed by independent full-text review of all potentially eligible records by the three reviewers. No automation tools were used during the screening process. Any disagreements were discussed, and the final study inclusions were agreed upon.

### 2.3. Data Extraction

A standardized data extraction form was developed in Microsoft Excel. Three reviewers independently extracted data from each included study. Extracted variables included study characteristics (study design and follow-up duration), participant characteristics (sample size, number of participants evaluated at follow up, mean age, mean body mass index [BMI], SUI type [pure or mixed], and prior treatment), intervention characteristics (stem cell source, cell dose, injection approach, procedural guidance modality, and use of post-procedural physiotherapy), study-defined response criterion, number of participants meeting the response criterion, and safety outcomes including overall biopsy-related and injection-related adverse event rates, severe adverse event rates, and specific complications (e.g., urinary tract infection, irritative lower urinary tract symptoms, and voiding dysfunction). For RCTs, comparator type, intervention- and control-arm sample sizes, numbers of responders in each group, outcome definition, and assessment time point were extracted. Discrepancies between reviewers were resolved through discussion and consensus.

### 2.4. Quality Assessment

Risk of bias was assessed independently by three reviewers using tools appropriate to the study design and the evidence contributed to the review. Randomized controlled trials evaluating cell-based therapy against a control or comparator intervention were assessed using the Cochrane Risk of Bias 2 (RoB 2) tool for the relevant efficacy outcome. The five RoB 2 domains included bias arising from the randomization process, deviations from intended interventions, missing outcome data, measurement of the outcome, and selection of the reported result [25]. Overall judgments were categorized as low risk of bias, some concerns, or high risk of bias. Prospective uncontrolled studies were assessed using the Joanna Briggs Institute (JBI)’s Critical Appraisal Checklist for Case Series [26]. The certainty of evidence for the principal outcomes was assessed using the Grading of Recommendations Assessment, Development and Evaluation (GRADE) approach, considering risk of bias, inconsistency, indirectness, imprecision, and other relevant considerations [27]. The traffic-light plot was generated using robvis [28] and is presented as Figure S1 in the Supplementary Materials.

### 2.5. Statistical Analysis

Statistical analyses were performed using Stata 19.5 (StataCorp LLC, College Station, TX, USA). Post-treatment response proportions in cell-treated cohorts were pooled using the Freeman–Tukey double-arcsine transformation and subsequently back-transformed for presentation. Random-effects models were fitted using restricted maximum likelihood (REML) estimation to account for anticipated between-study heterogeneity arising from differences in patient characteristics, stem cell type, procedural technique, and outcome definitions. Statistical heterogeneity was quantified using the I^2^ statistic. Small-study effects were explored visually using a funnel plot and statistically using Egger’s regression test. Robustness of the primary pooled estimate was examined using leave-one-out sensitivity analysis.

Because treatment success was defined inconsistently across studies, each study was categorized according to its reported response definition to allow exploration of its potential impact on the measured treatment response. Responses based on pre-specified objective thresholds for improvement (e.g., ≥50% reduction in incontinence episode frequency on a voiding diary or pad test, or a negative cough stress test) were classified as objective-threshold responses. Responses based on patient-reported improvement were classified as subjective response. Studies reporting only aggregate continuous outcome measures (e.g., mean pre- and post-treatment pad weight, incontinence episode frequency, or questionnaire scores, etc.) without reporting the number of participants meeting a defined response criterion were not included in the pooled post-treatment response analysis. These studies were synthesized narratively.

The primary analysis estimated the pooled post-treatment response proportion among women receiving stem cell-based injection therapy for SUI, reported as percentages with corresponding 95% confidence intervals. To investigate potential sources of heterogeneity, subgroup analyses were performed according to the study-defined response category, cell-source category, SUI type (pure SUI vs. mixed urinary incontinence [MUI]), use of ultrasound guidance, injection approach (transurethral vs. periurethral), and use of post-treatment physiotherapy, where sufficient data were available. Univariable meta-regression analyses were conducted for continuous moderators, including stem cell dose, mean age, and BMI, where sufficient studies were available. All subgroup and meta-regression analyses were considered exploratory because they were based on study-level characteristics and relatively small numbers of studies within some categories. Comparative evidence from RCTs was analyzed separately from the pooled post-treatment response analysis. Placebo-controlled trials reporting comparable response outcomes at similar follow-up time points were pooled using risk ratios (RRs) with 95% confidence intervals. RCTs which were not sufficiently comparable for quantitative synthesis were summarized narratively.

Safety outcomes were synthesized descriptively because adverse-event reporting was not sufficiently standardized for quantitative pooling.

## 3. Results

The systematic database search retrieved 1967 records. Hand-searching of reference lists from the included studies and relevant review identified nine records. Following deduplication, 1729 unique records remained and were screened at the title and abstract level, of which 1639 records were excluded as they did not include specifically SUI studies, or the specified intervention. The 90 remaining records were retrieved for full-text assessment, from which a further 69 were excluded for the following reasons: animal or preclinical studies only (*n* = 31), review articles, book chapters, editorials, or correspondence (*n* = 28), male-only populations or mixed-sex cohorts from which female-specific outcomes could not be isolated (*n* = 2), no available English translation (*n* = 1), retracted publication (*n* = 1), non-stem cell injections (*n* = 3), and duplicate reporting from the same patient cohort as an included study (*n* = 4). A total of 21 studies met the eligibility criteria and were included in the review. The study selection process is illustrated in the PRISMA flow diagram (Figure 1).

### 3.1. Studies Characteristics

The twenty-one studies included in this review were published between 2007 and 2026. They enrolled a combined total of 885 female patients across 15 countries spanning Europe, North America, Middle East and Asia. The United States and Iran contributed five studies each, with the remainder originating from UK, Canada, South Korea, Slovenia, Spain, Switzerland, Finland, Poland, France, Germany, Austria, and Brazil [29,30,31,32,33,34,35,36,37,38,39,40,41,42,43,44,45,46,47,48,49]. Sample sizes ranged from 5 to 199 patients (median = 15). The small sample size in most studies reflects the predominantly early-phase nature of the evidence base.

Fifteen studies evaluated MDSCs or myoblasts [29,30,31,32,33,34,35,36,37,38,39,40,41,42,43], three evaluated adipose-derived mesenchymal stem cells (ADSCs) [44,45,46], one evaluated oral mucosa-derived stem cells [47], one evaluated umbilical cord blood-derived stem cells [48], and one evaluated MDSCs and bone marrow-derived stem cells [49]. All cell preparations were autologous in origin except in Lee et al. (2010) [48], which used allogenic umbilical cord blood-derived stem cells. Across studies reporting cell dose, administered quantities spanned a wide range from 200,000 to 430,000,000 cells. A transurethral approach was utilized in thirteen studies [29,30,32,33,34,36,37,38,39,41,44,45,48], four studies used a periurethral injection approach [31,43,47,49], and four studies used both approaches [35,40,42,46]. Six studies utilized ultrasound guidance [30,31,32,33,34,35]. Post-procedural physiotherapy was administered in four studies post-therapy [30,32,33,34].

The predominant patient profile across the included literature was refractory SUI. Sixteen studies enrolled patients who had failed at least one prior treatment [29,31,32,34,36,37,38,39,40,41,42,43,44,45,47,48]. Six studies included patients with MUI or an urgency component associated with the incontinence [29,36,40,45,48,49]. Follow-up duration ranged from 3 to 48 months, with seventeen studies reporting outcomes at 12 months or more, see Supplementary Table S1. Mean patient age across studies ranged from 45 to 63 years. Key characteristics of all included studies are summarized in Supplementary Table S1.

Risk-of-bias assessments, according to study design, are presented in Supplementary Table S2A,B and Figure S1. Among the five comparative RCTs, Kaufman et al. and Jankowski et al. were judged to have low overall risk of bias [29,36]; Daneshpajooh et al. and Mahboubeh et al. were judged to have some concerns [43,47]; and Rose and Rübben was judged to have high risk of bias [30]. The main concerns related to unclear allocation concealment or insufficient information to verify prespecified analyses in the smaller trials, and to open treatment allocation, post-randomization exclusions, and participant-reported outcome assessment in Rose and Rübben [30]. Schmid et al. was not included in the comparative RoB 2 assessment because all participants received cell therapy and randomization concerned only the addition of neuromuscular electromagnetic stimulation [33]. The cell-treatment evidence from this study was therefore appraised with the prospective uncontrolled studies using the JBI checklist. Among the prospective uncontrolled studies [31,32,33,34,35,37,38,39,40,41,42,44,45,46,48,49], JBI appraisal showed a generally adequate reporting of eligibility criteria, diagnosis, participant characteristics, and outcomes. The most frequent limitation was unclear or non-consecutive recruitment, with occasional uncertainty regarding complete inclusion and outcome reporting. Detailed study-level assessments are provided in Supplementary Table S2B. The certainty of evidence for the principal outcomes was assessed using the GRADE approach, with the detailed assessment provided in Supplementary Table S2C [27].

### 3.2. Post-Treatment Response

Seventeen studies were included in the primary analysis of post-treatment response. Four studies (Blaganje et al. 2022 [32]; Schmid et al. 2023 [33]; Dutta et al. 2025 [35]; and Arjmand et al. 2017 [46]) were excluded from the pooled analysis because they reported only pre- and post-treatment mean value comparisons without providing the data necessary for meta-analytic pooling. Using a random-effects meta-analysis, the pooled post-treatment response proportion was 63.7% (95% CI 56.7–70.5%). Heterogeneity was moderate to substantial (I^2^ = 62.6%, *p* < 0.001). Individual cohort response proportions ranged from 47.0% to 79.0%. The largest treatment cohorts reported response proportions of 52.5% in Kaufman et al. 2024 [29], 77.8% in Rose and Rübben 2025 [30], 57.1% in Jankowski et al. 2018 [36], and 79.0% in Mitterberger et al. 2007 [34], see Figure 2. Visual inspection of the funnel plot did not show marked asymmetry; see Figure 3. Consistent with this finding, the regression-based Egger test provided no statistical evidence of small-study effects (β_1_ = −0.47, standard error = 0.691; z = −0.68; *p* = 0.496).

#### 3.2.1. Subgroup Analysis

The pooled response proportion was 63% (95% CI 55–70%; I^2^ = 65.4%) among studies that used objective threshold-based response definition and 74% (95% CI 58–88%; I^2^ = 72.4%) among studies that defined response using patient-reported outcomes. The between-subgroup difference was not statistically significant (*p* = 0.21).

The pooled response proportion differed according to the use of ultrasound guidance. Across three ultrasound-guided cohorts (*n* = 258), the pooled proportion was 78% (95% CI 73–83%). In 14 cohorts that did not use ultrasound guidance, the pooled proportion was 57% (95% CI 51–62%). The between-subgroup difference was statistically significant (*p* < 0.001).

The pooled response proportion was also higher in cohorts restricted to women with pure SUI than in cohorts that included women with mixed urinary incontinence. The pooled estimate was 76% (95% CI 70–81%) among nine pure-SUI cohorts and 55% (95% CI 48–62%) among six mixed-incontinence cohorts. The between-subgroup difference was statistically significant (*p* < 0.001).

Two cohorts that incorporated post-treatment physiotherapy (*n* = 236) had a pooled proportion of 78% (95% CI 73–84%), compared with 58% (95% CI 52–63%) across 15 cohorts without post-treatment physiotherapy. The between-subgroup difference was statistically significant (*p* < 0.001).

There was no statistically significant difference according to the cell-source category. The pooled proportion was 63% (95% CI 55–71%) in 13 cohorts using muscle-derived cells and 67% (95% CI 56–78%) in five cohorts using non-muscle-derived cells (*p* = 0.58). Similarly, injection approach was not a statistically significant moderator. The pooled response proportion was 66% (95% CI 57–74%) in 11 cohorts using transurethral injection and 64% (95% CI 52–75%) in four cohorts using periurethral injection (*p* = 0.76). The subgroup analysis is summarized in Figure 4.

#### 3.2.2. Meta-Regression

In univariable meta-regression, the mean administered cell dose was not significantly associated with post-treatment response (β = −0.00007, 95% CI −0.0013 to 0.0012; *p* = 0.91). Mean participant age was not significantly associated with post-treatment response (β = 0.0037, 95% CI −0.0193 to 0.0266; *p* = 0.75). Similarly, mean BMI was not significantly associated with post-treatment response (β = −0.0127, 95% CI −0.0477 to 0.0223; *p* = 0.48).

#### 3.2.3. Sensitivity Analyses

In the leave-one-out analysis, pooled estimates ranged from 61.6% to 65.3%. The lowest estimate was observed after the omission of Mitterberger et al. 2007 [34] and the highest after omission of Peters et al. 2014 [40]. The confidence intervals and pooled estimates remained broadly similar across all sequential omissions, indicating that no individual cohort materially influenced the primary result.

### 3.3. Comparative Evidence from Randomized Controlled Trials

Five RCTs provided comparative evidence between stem cell-based therapy and a non-stem cell control [29,30,36,43,47]. Two placebo-controlled RCTs reported the proportion of patients achieving ≥50% reduction in stress incontinence episode frequency at 12 months and were included in an exploratory comparative meta-analysis [29,36]. Both trials compared stem cell-based injection therapy with placebo injection. The pooled analysis showed no significant difference in response between the two groups (RR 1.02, 95% CI 0.85–1.23, *p* = 0.85), see Figure 5.

The remaining placebo-controlled RCT, Rose and Rübben, compared two cell-dose groups with oral duloxetine-placebo and duloxetine and therefore was not included in the pooled analysis [30]. The remaining two RCTs compared cell-based therapy with surgical treatment but used different surgical comparators and response outcomes. Therefore, their findings were synthesized narratively [43,47].

### 3.4. Safety and Complications Profile

Safety outcomes were reported in all 21 studies, although the scope and format of reporting varied substantially. Some studies reported the number of participants experiencing an adverse event, whereas others reported numbers of adverse-event episodes or provided only qualitative safety descriptions. Studies also differed in whether they reported all treatment-emergent events or only events considered related to the cell product, tissue-harvesting procedure, or injection. Accordingly, adverse events were synthesized by clinical category rather than combined as a simple aggregate count.

Overall, reported complications were predominantly minor, transient, and related to either tissue harvesting or injection. Irritative lower urinary tract symptoms were the most consistently documented injection-related events. Dysuria was the most frequently reported symptom, occurring in 14 of 199 women in the largest trial, seven of 80 women in the Peters et al. study, seven of 15 women assessed shortly after injection in the Souza et al. study, and four of 15 women in the Mirzaei study [29,40,47,49]. Urgency, frequency, pollakiuria, and urethral burning were reported less often and generally resolved spontaneously or with conservative treatment. In the Souza et al. cohort, none of the early irritative symptoms persisted at 12 months [49].

Urinary tract infection or cystitis was also reported across several studies but usually affected only a small proportion of treated women and typically resolved with antibiotic therapy [29,30,31,32,33,35,36,39,40,47]. The largest trial reported six UTIs among 199 treated women, while two UTIs were reported among 117 cell-treated women in the Rose and Rübben study and one among 80 women in the Peters et al. study [29,30,40]. Other studies reported isolated cases or small numbers of infections, whereas several cohorts explicitly reported no UTI following treatment [34,37,38,42,43,45,48,49].

Souza et al. reported injection-site pain in six of 15 evaluated women and bleeding, ecchymosis, or hematoma in nine [49]. In the largest trial, injection-site pain and hemorrhage were reported in 11 and five of 199 women, respectively [29]. Peters et al. additionally reported pelvic or abdominal pain in four of 80 women and transient hematuria in two [40].

Complications related to tissue harvesting consisted mainly of local pain, tenderness, bruising, or hematoma. Souza et al. reported biopsy-site pain in 11 of 19 evaluated women and bleeding, ecchymosis, or hematoma in four [49]. Peters et al. reported biopsy-related events in four participants, including wound hematoma, procedural dizziness, postoperative bleeding requiring suturing, and joint swelling [40]. Carr et al. reported pain or bruising in three of 38 women and one vasovagal episode, while four of ten women in the Sharifiaghdas cohort experienced biopsy-site pain [31,39]. No serious tissue-harvesting complication was reported.

Voiding dysfunction and urinary retention were uncommon and generally transient. Carr et al. reported mild self-limiting urinary retention in two of 38 women [31], while Souza et al. reported one case of voiding dysfunction among ten evaluated skeletal muscle-derived cell recipients and none among five bone marrow-derived cell recipients [49]. In the Mitterberger study, 12 of 123 women required temporary postoperative catheterization, although the investigators did not classify this as a complication [34].

Serious treatment-related adverse events were rare. One of 199 treated women experienced a serious hypersensitivity reaction that resolved within 24 h [29]. Carr et al. reported two additional transient allergic reactions that did not require intervention [31]. No treatment-related deaths were reported [29,30,31,32,33,34,35,36,37,38,39,40,41,42,43,44,45,46,47,48,49].

## 4. Discussion

Overall, the findings suggest that stem cell-based therapies have a generally favorable short- to medium-term safety profile and are associated with improvements in objective, patient-reported, and urodynamic outcomes. However, the therapeutic efficacy of these interventions remains uncertain. Most of the evidence was derived from small, uncontrolled studies, and the available randomized trials have not consistently demonstrated superiority over placebo or established surgical treatments. Accordingly, the pooled response estimates should be interpreted as proportions of women who improved after treatment rather than as estimates of treatment efficacy attributable to the cellular intervention itself.

### 4.1. Factors Associated with Post-Treatment Response

Subgroup analyses identified ultrasound-guided injection and restriction of enrollment to women with pure SUI as factors associated with higher pooled post-treatment-response proportion. Studies using real-time ultrasound guidance reported higher response proportion than studies using non-guided injection techniques. Ultrasound guidance may facilitate more accurate intra-sphincteric delivery, reduce variability in injection depth and distribution, and increase the likelihood that an adequate proportion of the administered cells reaches the intended anatomical target. Nevertheless, no included study directly randomized participants to ultrasound-guided versus non-guided injection; therefore, a causal effect of ultrasound guidance cannot be established.

Interpretation of this association is further complicated by the overlap between injection guidance and patient phenotype in the included studies. All studies using ultrasound guidance enrolled predominantly women with pure SUI, whereas several non-guided studies included women with mixed urinary incontinence (MUI). However, when the analysis was restricted to studies of pure SUI, ultrasound guidance remained significantly associated with post-treatment response. Ultrasound-guided procedures achieved a pooled response proportion of approximately 78% (95% CI 73–83%; I^2^ < 1%) compared with 65% (95% CI 53–76%; I^2^ < 1%) for non-guided procedures (*p* = 0.019). This finding suggests that the observed association was not explained solely by differences in SUI subtype. However, residual confounding by other study-level characteristics cannot be excluded.

Pure SUI was also associated with a higher pooled post-treatment response proportion than MUI in the initial subgroup analysis. The lower response observed in studies including women with MUI may reflect the contribution of urgency symptoms or detrusor overactivity, which would not be expected to respond directly to an intervention targeting urethral sphincter structure or function. However, among studies in which ultrasound guidance was not used, response rates were comparable between predominantly pure-SUI and MUI cohorts. This suggests that the apparent association between SUI subtype and treatment response may be partly explained by differences in delivery technique or other study characteristics. Given the limited number of studies in these subgroup analyses, both findings should be regarded as hypothesis-generating and investigated prospectively using well-characterized patient populations.

Post-treatment physiotherapy was associated with a higher pooled response proportion, with estimates of 78% in cohorts incorporating physiotherapy and 58% in those without it. This finding is biologically plausible, as pelvic floor muscle training may improve pelvic floor function and contribute to the observed post-treatment response. However, only two cohorts contributed to the physiotherapy subgroup, and these cohorts also shared other potentially favorable characteristics, including ultrasound-guided cell delivery. The observed association may therefore reflect confounding by procedural or study-level factors rather than an independent effect of physiotherapy.

The relationship between cell dose and post-treatment response remained unclear. Mean administered cell dose was not significantly associated with response in univariable meta-regression. These findings do not support a consistent study-level dose–response relationship. This interpretation is consistent with the mixed dose–response patterns reported by different studies. Peters et al. reported greater improvements in stress leakage and pad-test outcomes at higher doses (100–200 × 10^6^ cells) and observed better outcomes with two administrations of 32 × 10^6^ cells than with single lower-dose injections [40]. In contrast, Sèbe et al. found no clear dose–response relationship across doses of 10–50 × 10^6^ muscle-derived cells [41], while Rose and Rübben reported no significant difference between low- and high-dose cohorts using ultrasound-guided intra-sphincteric delivery [30]. Taken together, these findings suggest that the number of cells administered alone may not determine treatment response. The biologically effective dose may also depend on factors such as delivery precision, cell viability and processing, and the proportion of cells reaching and remaining within the target tissue [50,51,52].

### 4.2. Clinical Outcomes and Comparative Randomized Evidence

Reductions in pad weight and incontinence episode frequency were reported following treatment, although substantial variation was observed across studies. Mean reductions in pad weight ranged from 2.7 to 63.7 g [29,30,31,32,35,39,40,41,44,45,49]. Larger absolute reductions were generally observed in cohorts with greater baseline leakage, including the 63.7-g reduction reported by Sèbe et al. using a 24-h pad test [41]. Conversely, several studies enrolling women with lower baseline pad weights reported reductions of less than 10 g [32,40,44,49]. Direct comparison of these findings is limited by differences in pad-test protocols. The included studies used 20-min, 1-h, and 24-h tests, which measure leakage over substantially different observation periods and are not directly interchangeable [29,30,31,32,35,39,40,41,44,45,49]. Differences in baseline severity, testing instructions, bladder volume, physical activity protocols, and follow-up duration may also have contributed to the variability in reported changes. Therefore, the absolute reductions in pad weight should not be interpreted as representing a uniform treatment effect across studies. Incontinence episode frequency, assessed using 3-day or 7-day bladder diaries, was reported in seven cohorts [29,30,31,32,35,41]. All cohorts demonstrated numerical reductions after treatment, ranging from 3.5 to 17.4 episodes. As with pad weight, larger absolute reductions were generally observed in cohorts with higher baseline episode frequencies [30,32,41]. Individual-level results, when available, nevertheless demonstrated heterogeneous responses, with some participants achieving substantial improvement and others experiencing limited or no sustained benefit [29,30,31,41].

Patient-reported outcomes were assessed using several validated instruments, including the UDI-6, IIQ-7, and IQOL questionnaire. UDI-6 scores decreased in all three reporting cohorts, with mean reductions ranging from 3.2 to 27.7 points [31,35,45]. IIQ-7 scores also decreased, with reported reductions ranging from 2.9 to 23.8 points [31,35,45]. IQOL scores generally increased, indicating improved quality of life, with reported improvements ranging from 3.8 to 32.9 points [30,32,43,49], although some cohorts demonstrated only minimal changes [49]. These findings suggest that some women may experience meaningful symptomatic and quality-of-life improvement following treatment. However, interpretation is constrained by the small number of studies reporting each instrument, differences in baseline symptom severity, variable follow-up periods, and incomplete reporting of established minimal clinically important differences. The absence of blinded control groups in most studies also prevents differentiation between treatment-specific effects and improvements related to expectations, procedural context, concomitant interventions, or natural symptom fluctuation.

Maximum urethral closure pressure was reported in eight cohorts [32,33,34,37,38,39,44,48]. Most studies observed increases following treatment, ranging from 0.5 to 24.7 cmH_2_O [34,37,38,39,48], whereas others reported decreases of 7.9 to 16 cmH_2_O [32,33,44]. Considerable variation was also present in baseline MUCP values and post-treatment changes. The absence of a consistent urodynamic response weakens the proposition that the observed clinical improvements uniformly result from restoration of urethral sphincter function. Notably, Stangel-Wójcikiewicz et al. observed larger MUCP increases among women who achieved complete or partial clinical improvement than among nonresponders, suggesting some concordance between urethral pressure and clinical response; however, this was based on a small cohort, and no formal correlation analysis was performed [37]. In contrast, other studies demonstrated improvements in leakage, pad-test results, or patient-reported outcomes despite no significant increase in MUCP, and in some cases MUCP decreased [32,33]. These findings suggest that changes in MUCP may not directly parallel clinical improvement and that MUCP alone may be an incomplete marker of treatment response. Differences in urodynamic equipment, measurement protocols, bladder volume, and operator technique may also have contributed to the between-study variability.

The pooled placebo-controlled evidence did not demonstrate a treatment-specific benefit for the ≥50% response at 12 months. This substantially weakens the interpretation of the high response proportions reported in uncontrolled studies, as much of the observed improvement may reflect procedural, placebo, or other non-specific effects. However, the available evidence does not exclude smaller effects or effects in selected patient groups, and therefore does not establish that cell-based therapy is universally ineffective.

The largest available placebo-controlled trial was the multicenter phase III randomized controlled trial conducted by Kaufman et al., which enrolled 297 participants [29]. Despite its rigorous design, the trial did not demonstrate a statistically significant difference between the treatment and placebo groups in the primary efficacy endpoint of a ≥50% reduction in stress incontinence episode frequency at 12 months. This result was influenced by an unexpectedly high placebo response of 53.6%, compared with the 30% placebo-response rate assumed in the sample-size calculation. The high response in the control group illustrates a major methodological challenge in evaluating cell-based therapies for SUI. Interventions involving muscle biopsy, intra-sphincteric injection, repeated clinical assessments, and intensive follow-up may generate substantial treatment expectancy and contextual effects. In addition, the injection procedure itself may induce a transient bulking effect or local tissue response, even when no active cellular product is administered. Placebo responses in sham-controlled procedural trials may therefore exceed those usually observed in pharmacological studies [41].

A further randomized placebo-controlled study by Ismail et al. evaluated urethral sphincter morphometry using three-dimensional/four-dimensional trans-perineal ultrasonography as its primary endpoint in 58 women [53]. Both the autologous muscle-derived cell group and the placebo group demonstrated increases in total and external urethral sphincter volumes at 12 months. However, no significant between-group differences were observed in sphincter morphometry or stress incontinence episode frequency [53]. The improvement in both groups may indicate a tissue response related to needle injury or injection rather than a specific regenerative effect of the administered cells. The parallel changes in both groups raise the possibility that needle injury or the injection procedure itself contributed to the observed tissue response.

Together, these placebo-controlled findings substantially temper the encouraging results of earlier single-arm studies, in which observed improvement cannot be separated from treatment-specific effects, regression to the mean, natural symptom variation, procedural effects, and placebo response.

Although the Kaufman et al. trial did not demonstrate superiority over placebo at 12 months, participants in the active-treatment group experienced continued reductions in stress incontinence episodes during the subsequent open-label follow-up [29]. The sustained response raises the possibility of a delayed biological effect; however, the absence of a concurrent control group during extended follow-up prevents the attribution of this improvement specifically to cellular regeneration.

Interestingly, in the prespecified subgroup of women with previous SUI surgery, a significantly greater proportion of those receiving cell therapy achieved a ≥75% reduction in stress incontinence episodes compared with placebo (40% vs. 16%, *p* = 0.037) [29]. A separate post hoc analysis showed that a ≥75% reduction in incontinence episodes corresponded to a clinically meaningful improvement of at least 10 points in I-QOL score, supporting the clinical relevance of this response threshold. Nevertheless, the subgroup finding should be interpreted cautiously, particularly given the absence of an overall treatment benefit in the primary analysis. It provides a rationale for prospective evaluation of cell therapy specifically in women with persistent or recurrent SUI after previous surgery.

The two small surgery-controlled RCTs could not be quantitatively pooled because they evaluated different cell products, different sling procedures, and used different definitions of treatment response. The trials were therefore considered separately. In both studies, the reported clinical response measures generally favored sling surgery.

Daneshpajooh et al. randomized 30 women with SUI refractory to conservative management to periurethral muscle-derived stem-cell injection or mid-urethral sling surgery [43]. At 12 months, overall improvement was reported in 93.3% of women undergoing sling surgery compared with 66.6% receiving cell therapy, while complete recovery occurred in 80% and 33.3%, respectively. Although both groups improved symptomatically, outcomes consistently favored sling surgery [43].

A small noninferiority trial comparing autologous oral mucosa-derived cell injection with mini-sling surgery also reported improvement in both groups [47]. A negative Marshall test was observed in 73% of the cell-therapy group and 80% of the mini-sling group, while patient satisfaction was similar at 6 months but favored the mini-sling group at 2 years. The mini-sling group experienced more grade II complications, including dyspareunia. However, the small sample size and limitations in the statistical support for noninferiority preclude concluding that cell therapy provides efficacy comparable with surgery.

In the context of established SUI management, cell-based injection therapy remains investigational. Supervised pelvic floor muscle training remains the recommended first-line treatment, while surgical treatment is considered in women with persistent bothersome SUI after conservative management [8,14]. Urethral bulking agents provide a less invasive alternative for selected women but may require repeat treatment and are generally less effective than established surgical procedures [8,14]. Duloxetine may be considered in selected patients, although its use is limited by adverse effects and it is not generally recommended as first-line therapy [8,14,15]. Direct comparison of cell-based therapy with these established approaches remains limited. The included evidence contained two small randomized trials comparing cell-based therapy with sling surgery [43,47] and one randomized trial incorporating duloxetine as a comparator [30], while no eligible studies directly compared cell therapy with pelvic floor muscle training or conventional urethral bulking agents. The available surgery-controlled trials generally favored sling procedures [43,47]; therefore, current evidence does not support replacing established conservative or surgical treatments with cell-based therapy.

### 4.3. Durability and Safety

Long-term evidence remains limited because most studies reported outcomes within 12–24 months. Nevertheless, the available follow-up data suggest that improvement may persist in some responders. Sèbe et al. reported sustained benefit for up to 72 months in three participants [41], whereas Stangel-Wójcikiewicz et al. described maintained clinical improvement at 24 months and persistent quality-of-life benefits during subsequent follow-ups, to 48 months [37,54]. Ismail et al. reported continued reductions in pad-test leakage through 24 months, and Rose and Rübben described sustained improvements in stress incontinence episode frequency and quality of life during extended follow-up [30,53].

Although a gradual and sustained response is biologically compatible with tissue repair or remodeling, the long-term evidence is derived largely from small cohorts, selected responders, or uncontrolled extensions and therefore does not establish persistence of a treatment-specific effect.

The pooled safety findings indicate that cell-based injection therapy is generally well tolerated. Reported adverse events were predominantly mild and transient. Donor-site events included localized pain, bruising, hematoma, and occasional superficial wound infection. Injection-related events included transient dysuria, urgency, urinary discomfort, and short-term voiding symptoms [29,30,31,32,33,34,35,36,37,38,39,40,41,42,43,44,45,46,47,48,49]. Only three serious adverse events were reported across the included studies, all within the large Kaufman et al. trial [29]. These included a self-limiting hypersensitivity reaction, temporary urinary retention managed with intermittent catheterization, and one insufficiently characterized event [29]. No deaths were attributed to treatment [29,30,31,32,33,34,35,36,37,38,39,40,41,42,43,44,45,46,47,48,49].

Despite these reassuring findings, the available evidence is insufficient to establish long-term safety because most studies were small and not powered to detect rare adverse events. The limited duration of follow-up also prevents exclusion of uncommon, delayed complications, including abnormal tissue proliferation, ectopic differentiation, fibrosis, or tumorigenic events [54,55].

### 4.4. Limitations

Several limitations should be considered. Most included studies were small, uncontrolled cohorts, so the pooled response proportions describe improvement after treatment rather than effects attributable specifically to cell therapy. Considerable clinical and methodological heterogeneity was present across patient populations, cell preparations and doses, injection techniques, concomitant interventions, follow-up periods, and definitions of treatment response, while differences in measurement and reporting prevented quantitative pooling of several continuous outcomes. Subgroup and meta-regression analyses were based on study-level data and relatively few studies in some categories; therefore, observed associations, particularly with ultrasound guidance and SUI subtype, remain susceptible to confounding and should be considered exploratory. The small number of cohorts precluded reliable multivariable meta-regression to adjust for correlated study-level characteristics. Although Egger’s test did not indicate significant small-study effects, publication bias and selective reporting cannot be ruled out, particularly given the limited number and heterogeneity of the included studies. Safety reporting was also inconsistent, and the generally small cohorts and limited follow-up reduce confidence in detecting uncommon or delayed adverse events.

### 4.5. Clinical Implications and Future Research

Based on the current evidence, stem cell-based injection therapy for female SUI should be considered experimental and should not be offered as routine clinical care outside appropriately governed clinical trials or specialist research settings. The available comparative evidence does not demonstrate superiority over placebo and suggests lower efficacy than established sling surgery, despite potentially lower procedural morbidity. Women with persistent or recurrent SUI after previous anti-incontinence surgery may represent a particularly relevant population for future investigation.

Further adequately powered, procedure-matched placebo-controlled trials are needed to determine whether the cellular product provides an incremental benefit beyond the injection procedure and other nonspecific effects. The trials should use standardized cell preparation and delivery protocols, predefined clinically meaningful outcomes (including both objective leakage measures and validated patient-reported outcomes), and long-term follow-up extending for at least five years. Direct randomized evaluation of ultrasound-guided versus non-guided delivery would be particularly valuable. More broadly, as with other emerging stem cell-based and reproductive regenerative technologies, translation into clinical practice should be accompanied by long-term safety surveillance and appropriate ethical and regulatory oversight [56,57].

## 5. Conclusions

Stem cell-based urethral injection therapy for female SUI is associated with encouraging post-treatment response rates and a generally favorable short- to medium-term safety profile. Exploratory subgroup analyses identified an association between ultrasound-guided delivery and higher post-treatment response, but this finding remains susceptible to study-level confounding. Additionally, the observed response cannot be attributed specifically to the cellular component. An exploratory meta-analysis of two placebo-controlled RCTs did not demonstrate a higher 12-month response with cell therapy compared with placebo injection. The observed improvement may therefore reflect a combination of procedural, contextual, placebo, and potentially cellular effects, whose relative contributions remain uncertain. Surgery-controlled trials could not be quantitatively pooled, although their individually reported outcomes generally favored established sling procedures. Cell-based therapy should therefore remain investigational. Further adequately powered, procedure-matched placebo-controlled trials are needed to determine whether the cellular product provides benefit beyond the injection procedure and other nonspecific effects. These trials should use standardized treatment protocols, clinically meaningful outcomes, and adequate long-term follow-up.

## Figures and Tables

**Figure 1:**
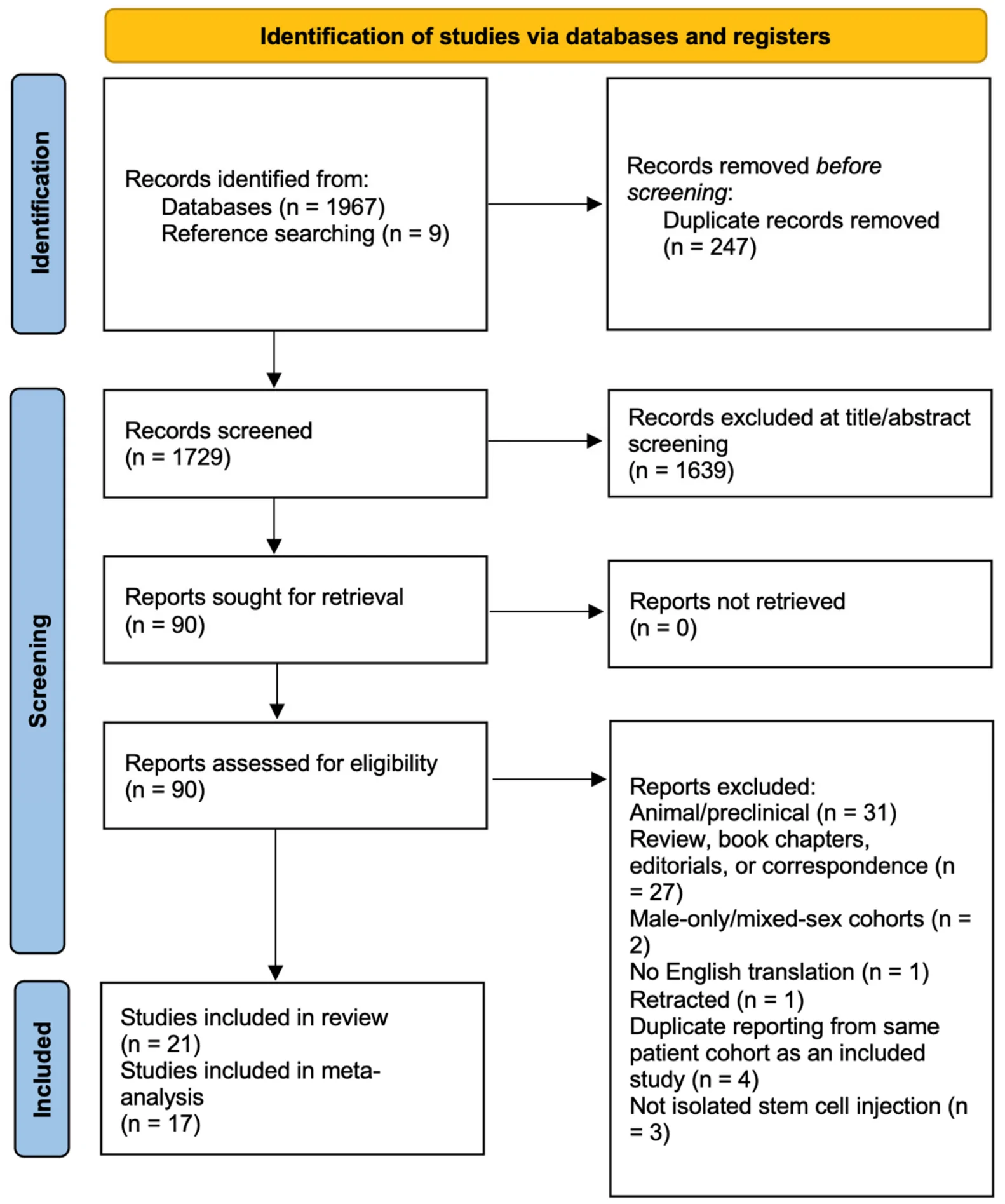
PRISMA 2020 flow diagram.

**Figure 2:**
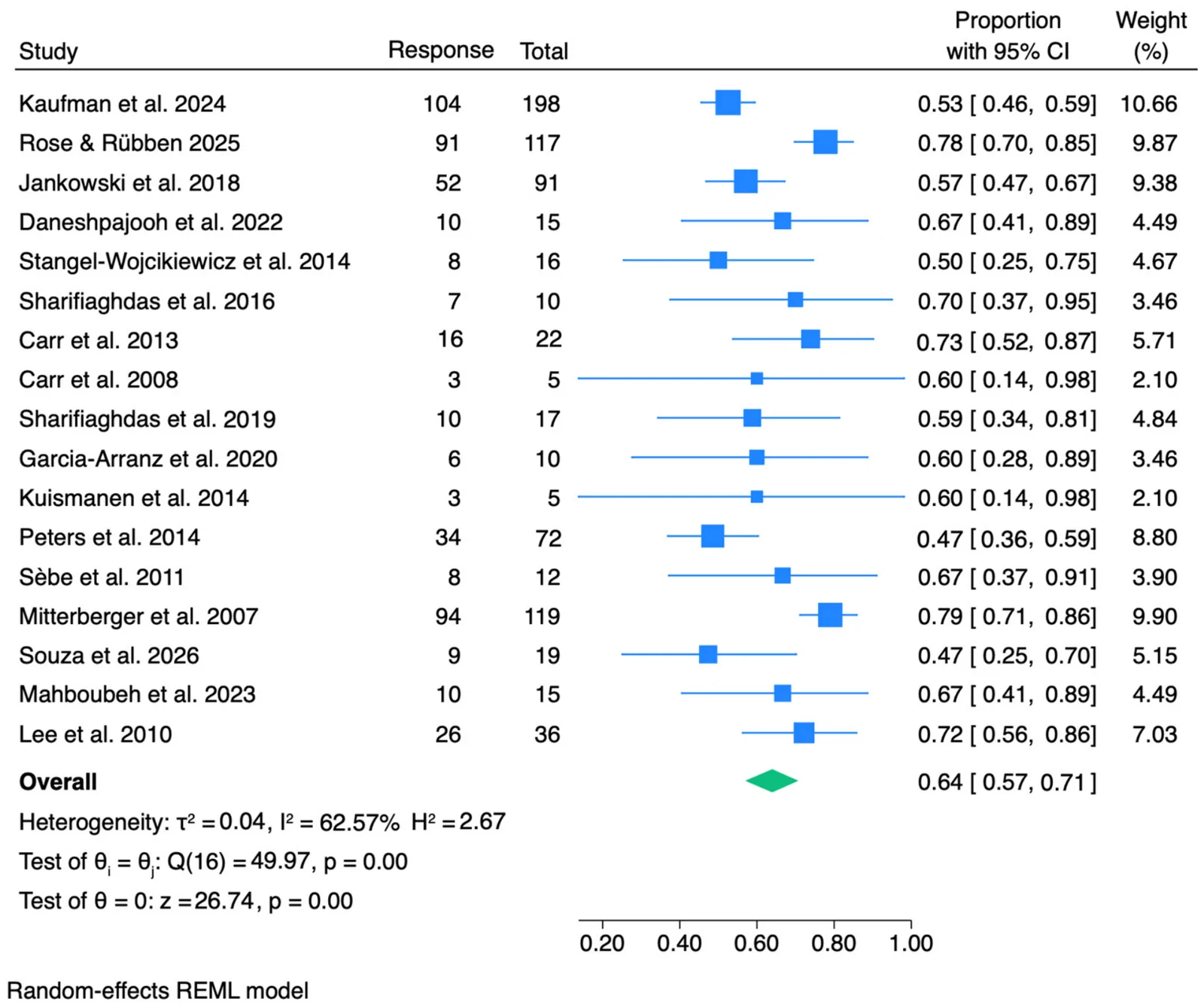
Forest plot of the overall post-treatment response proportion following stem cell-based therapy for female stress urinary incontinence, based on the included treatment cohorts [29,30,31,34,36,37,38,39,40,41,42,43,44,45,47,48,49]. Squares represent individual cohort estimates, with their size proportional to study weight; horizontal lines indicate 95% confidence intervals. The diamond represents the pooled response proportion.

**Figure 3:**
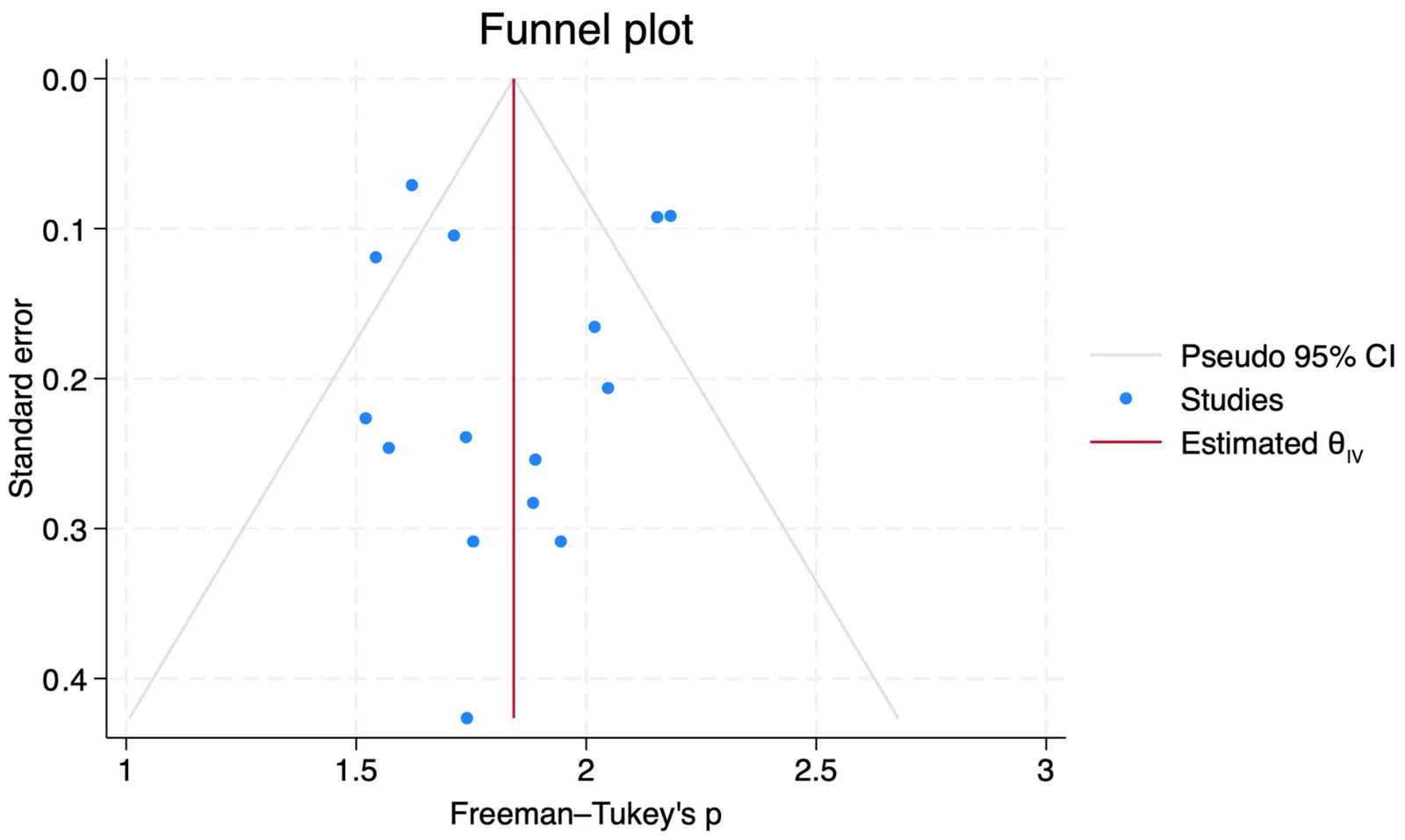
Funnel plot assessing publication bias for studies evaluating the post-treatment response of stem cell therapy in female stress urinary incontinence.

**Figure 4:**
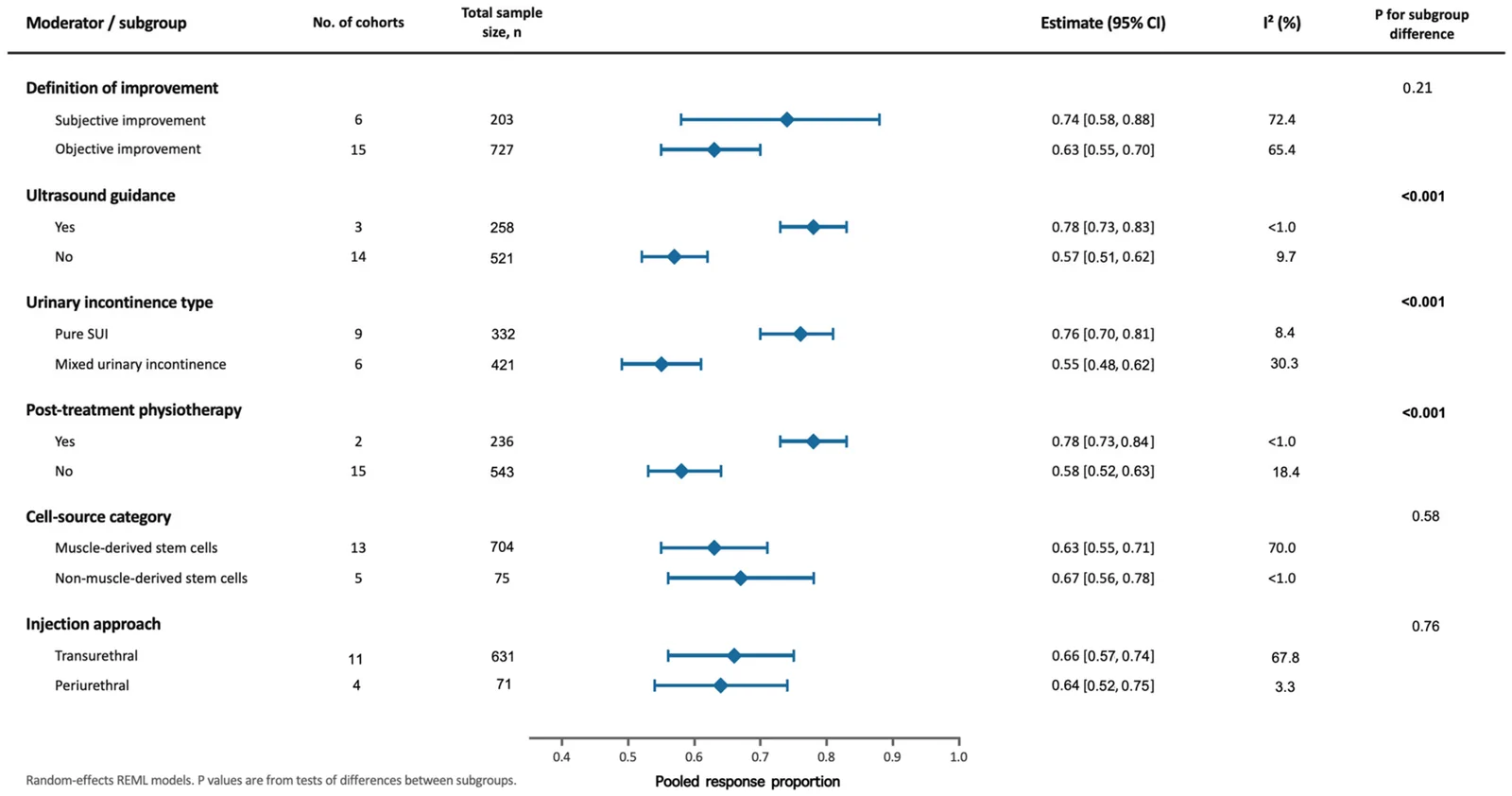
Summary forest plot of subgroup analyses of post-treatment response following stem cell-based therapy for female stress urinary incontinence. Squares and horizontal lines represent the pooled response proportions and corresponding 95% confidence intervals for each subgroup, respectively. *p* values represent tests of differences between subgroup categories. Estimates were calculated using random-effects model. The number of cohorts contributing to each subgroup varied according to data availability; individual studies could contribute more than one cohort where relevant to the subgroup classification.

**Figure 5:**
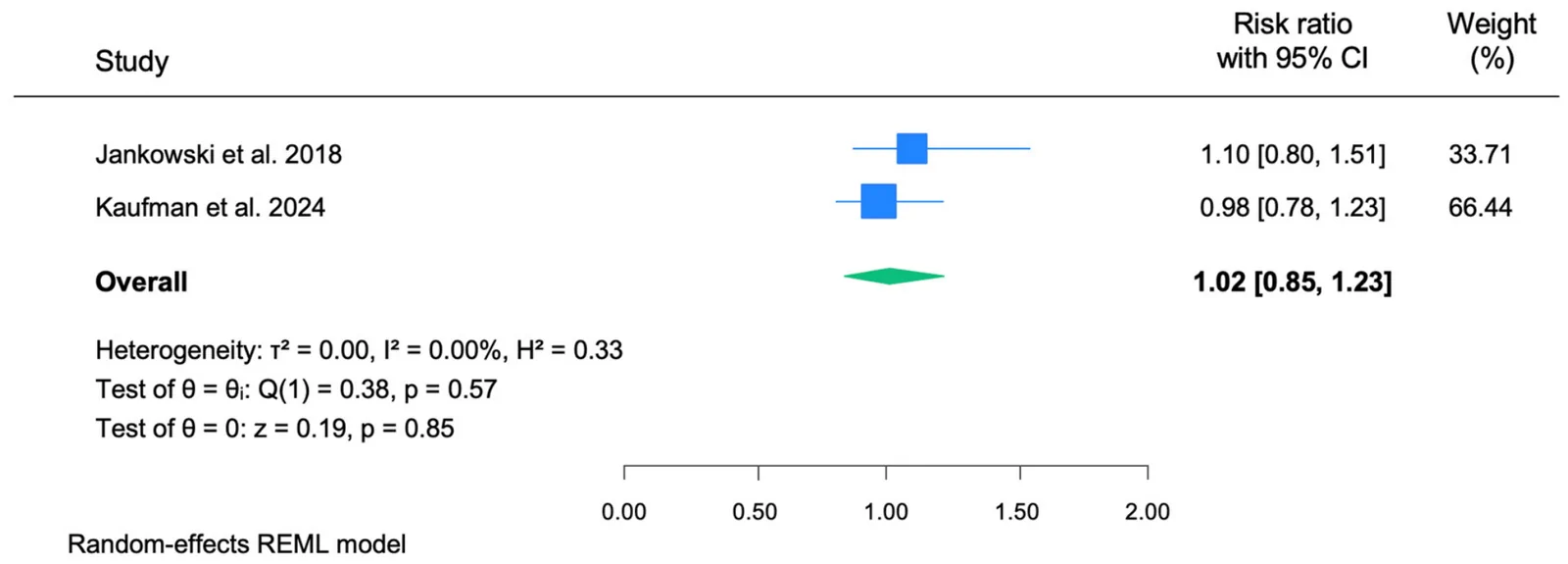
Comparative exploratory meta-analysis of placebo-controlled randomized trials reporting ≥50% reduction in stress incontinence episode frequency at 12 months [29,36]. Risk ratios greater than 1 favor cell-based therapy. Squares represent individual cohort estimates, with their size proportional to study weight; horizontal lines indicate 95% confidence intervals. The diamond represents the pooled effect size.

## Data Availability

Data is contained within the article or Supplementary Materials.

## Supplementary Materials

The following supporting information can be downloaded at: https://www.mdpi.com/article/10.3390/jcm15197737/s1, Table S1: Summary of Included Studies Evaluating Stem Cell-Based Therapy for Female Stress Urinary Incontinence; Table S2A: Risk-of-Bias Assessment of Randomized Controlled Trials Using Cochrane RoB 2 [25]; Table S2B: Critical Appraisal of Prospective Uncontrolled Studies Using the JBI Critical Appraisal Checklist for Case Series [26]; Table S2C: GRADE Summary of Findings and Certainty of Evidence for stem cell–based urethral injection therapy in women with stress urinary incontinence [27]; Table S3: PRISMA 2020 Checklist; Figure S1: RoB 2 Traffic-Light Plot [28].

## Abbreviations

The following abbreviations are used in this manuscript:

ADSCs	Adipose-derived stem cells
BMI	Body mass index
BMSCs	Bone marrow-derived mesenchymal stem cells
CI	Confidence interval
EAU	European Association of Urology
ICIQ-UI SF	International Consultation on Incontinence Questionnaire–Urinary Incontinence Short Form
ICS	International Continence Society
IIQ-7	Incontinence Impact Questionnaire–7
I-QOL	Incontinence Quality of Life Questionnaire
ISD	Intrinsic sphincter deficiency
JBI	Joanna Briggs Institute
MDSCs	Muscle-derived stem cells
MeSH	Medical Subject Headings
MUCP	Maximum urethral closure pressure
MUI	Mixed urinary incontinence
MUS	Mid-urethral sling
PRISMA	Preferred Reporting Items for Systematic Reviews and Meta-Analyses
PROSPERO	International Prospective Register of Systematic Reviews
RCT	Randomized controlled trial
RR	Risk Ratio
SUFU	Society of Urodynamics, Female Pelvic Medicine and Urogenital Reconstruction
SUI	Stress urinary incontinence
UDI-6	Urogenital Distress Inventory–6
UK	United Kingdom
USA	United States of America
UTI	Urinary tract infection
