# Supplementary material for: Post-Treatment Response and Safety of Stem Cell-Based Urethral Injection Therapy for Female Stress Urinary Incontinence: A Systematic Review and Meta-Analysis
Source: J Clin Med. 2026 Oct 8;15(19):7737. doi: 10.3390/jcm15197737 (PMC13665857; doi:10.3390/jcm15197737)
Supplement: Supplementary file 1 [file jcm-15-07737-s001.zip › jcm-4544245-supplementary.pdf]

Table S1. Summary of Included Studies Evaluating Stem Cell-Based Therapy for Female Stress Urinary Incontinence.

First author year [Ref]	Country	Age (years)	BMI (kg/m2)	SUI Diagnosis	N (treated)	Stem cell type	Dose (x 10^6 cells)	Follow-up time (month)	Outcome Measure	Pre- vs. post-treatment (mean ± SD or median [IQR]) [if reported]
Kaufman et al.2024* [29]	USA, Germany, UK	54.1 ± 10.9	26.9 ± 4.3	≥3 stress incontinence episodes in a 3-day bladder diary, symptoms for >6 months, and urethral mobility ≤30° on Q-tip testing	199	AMDSC	150 (± 20%)	12	• 3-day bladder diary<br>• 24-h pad test	• Significant reduction in IEF compared with baseline in both treatment group = −5.8 ± 11 ($p < 0.0001$), and placebo = −4.9 ± 13.5 ($p = 0.0006$), although the reduction was not statistically different between the groups
Rose & Rübben 2025* [30]	Bulgaria	55.0 ± 12.4	28.1 ± 5.8	Clinical symptoms, Hahn and Fall short pad test, and urodynamic testing	117	AMDSC	10 – 40	3	• 1 week bladder diary<br>• 1-h pad test<br>• IQoL	• Significant reduction in IEF = -17.4 ± 16.0<br>• Significant reduction in pad weight = - 11 ± 8.6 g<br>• No statistically significant change in IQoL scores
Carr et al. 2013 [31]	Canada	50.0 ± 2	25.9 ± 0.6	Clinical evaluation, cough and Valsalva stress tests, pad testing, bladder diary, and urodynamic confirmation	38	AMDSC	Low dose = 1 – 16<br>High dose = 32 – 128	18	• 3-day bladder diary<br>• 1-h pad test<br>• UDI-6	• Significant reduction in IEF: 7.0 ± 1.3 to 2.9 ± 0.7 (low dose), 3.9 ± 0.9 to 0.4 ± 0.2 leaks (high dose)<br>• Significant reduction in pad weight: 15.5 ± 7.7 to 12.8 ± 5.9 g (low dose), 15.7 ± 5.5 vs 2.0 ± 1.0 g (high dose)<br>• Significant reduction in UDI-6 score: 45.8 ± 3.8 to 34.2 ± 3.8 (low dose), 51.0 ± 5.2 vs 23.3 ± 5.9 (high dose)
Blaganje et al.	Slovenia	55.8 ±	24.6	SUI symptoms	38	AMDSC	0.2	12	• IEF score	• Significant reduction in IEF episodes: 22.0

2022 [32]		10.7		and urodynamic confirmation					• 20-minute pad test<br>• IQoL<br>• MUCP	[15.0–32.0] vs 9.0 [4.0–22.0], p <0.001<br>• Significant improvement of pad test (ml): 14.5 [4.0–36.0] vs 1.0 [0.0–7.0], p = 0.001<br>• Significant improvement of IQoL: 30.68 [18.18 – 38.64] to 60.23 [36.36 – 85.23], p<0.001<br>• Significant reduction of MUCP (cmH2O): 47.5 [38.0 – 75.0] to 43.5 [33.0 – 53.0], p<0.001
Schmid et al. 2023 [33]	Switzerland	45.67 ± 9.19	24.49 ± 3.04	SUI symptoms, standardized 1-h pad test, and urodynamic testing	9	AMDSC	80–100	6	• ICIQ-UI-SF	• Significant reduction of ICIQ-UI-SF score: 7 vs 4 points (p=0.035)
Mitterberger et al. 2007 [34]	Austria	62.8 ± 10,5	NR	Urodynamically confirmed SUI	123	AMDSC	38 fibroblasts and 28 myoblasts	12	• 24-h bladder diary<br>• 24-h pad test<br>• IQoL	• Significant reduction in IEF: 6 vs 0 (p= 0.001)<br>• Significant improvement of IQoL score: 51 vs 108 (p= 0.001)<br>• Rhabdosphincter thickness increased from 2.1 ±0.3 mm to 3.4 ± 0.4 mm (p = 0.001)<br>• MUCP during voluntary contraction improved from 51.7 ± 35.8 cmH2O to 78.1 ± 56.2 cmH2O (p = 0.001)
Dutta et al. 2025 [35]	USA	53.4 ± 9.6	31.0 ± 4.9	Clinical evaluation and urodynamic testing; exact diagnostic criteria not reported	8	AMDSC	1 – 128	12	• 1 week bladder diary<br>• 1-h pad test<br>• UDI-6	• Reduction in IEF: 15.3 ± 15.7 to 8.1 ± 15.4 leaks (p = 0.06)<br>• Reduction in 1-h pad weight: 39.3 ± 55.5 to 3.8 ± 8.72 g (p < .0001)<br>• Reduction in UDI-6 score: 8.6 ± 3.9 to 5.4 ± 4.0 (p = 0.003)
Jankowski et al. 2018* [36]	Canada, UK, Germany	51.4 ± 11.1	26.3 ± 4.4	≥3 stress incontinence episodes in a 3-day bladder	93	AMDSC	150 – 300	12	• 3-day bladder diary<br>• 24-hour	• Treatment and placebo groups had similar improvement from baseline in subject-reported symptom severity, as assessed by IQoL, IIQ-7, GQoL, UDI-6, and ISI score (p

				diary, symptoms for >6 months, and observed leakage during focused stress-incontinence evaluation					pad tests<br>• QoL	> 0.05)
Stangel-Wójcikiewicz et al. 2014 [37]	Poland	56.75 ± 7.63	26.05 ± 6.94	Clinical stress testing, including CLPP and VLPP, and urodynamic testing	16	AMDSC	0.6–25	8	• CLPP and VLPP Stress Tests<br>• Gaudenz Questionnaire<br>• MUCP	• Statistically significant increase of mean MUCP from baseline after treatment from 28.4 ± 17.8 to 45.6 ± 25.4 cmH2O
Sharifiaghdas et al. 2019 [38]	Iran	51 ± 10	NR	Clinical evaluation, cough stress and pad tests	20	AMDSC	50	12	• Cough stress test<br>• 1-h pad test<br>• MUCP<br>• UDI-6, IIQ-7	• No change observed in the mean MUCP from baseline at 24 months.<br>• Significant reduction in the mean UDI-6 and IIQ-7 scores, from 11 to 8 and 12.5 to 8 respectively ($p < 0.001$)
Sharifiaghdas et al. 2016 [39]	Iran	29.2 ± 15.3	NR	Clinical evaluation, cough stress and pad tests, and urodynamic testing	10	AMDSC	25	12	• Cough stress test<br>• 1-h pad test<br>• MUCP<br>• IIQ-7	• Significant decrease in the mean 1-h pad weight from 49.1 (31-67) g to 23.7 (0-52) g at 12 months<br>• Significant increase in the mean MUCP from 15.6 (9-25) cmH2O to 26.1 (14-52) cmH2O at 12 months<br>• Significant reduction in mean IIQ-7 score from 45.6 (29.3-66.7) to 37.1 (13.1-63.7) at 12 months
Peters et al. 2014 [40]	USA Canada	55 ± 1	27.8 ±0.6	SUI symptoms for >6 months and urodynamic	80	AMDSC	10-200	12	• 3-day bladder diary<br>• 24-h pad	• Significant reduction in mean IEF in all dose groups at 12 months<br>• Significant reduction in mean pad weight was observed only in the 200x10^6 dose

				confirmation					tests<br>• UDI-6 & IIQ-7	group at 12 months.<br>• Mean UDI-6 and IIQ-7 scores decreased significantly, independent of dosage.
Sèbe et al. 2011 [41]	France	58±13.5	27.6±4.7	Clinical evaluation, 1-h pad test, 7-day bladder diary, and urodynamic testing	12	AMDSC	10-50	12	• 7-day bladder diary<br>• 1-h pad test<br>• CONTILIFE QoL	• Reduction in IEF: 62.6 ± 56.6 to 50.4 ± 48.2<br>• Reduction in pad weight: 97.8 ± 151.7 to 34.1 ± 47.8 g<br>• Increase in CONTILIFE QoL score: 2.92 ± 1.74 to 5.18 ± 2.61
Carr et al. 2008 [42]	Canada	54 (42-65)	NR	Clinical evaluation, baseline pad testing, and urodynamic confirmation	8	AMDSC	18–22	16.5	• Bladder diary<br>• Pad weight test	• NR
Daneshpajooh et al. 2022* [43]	Iran	48.9 ± 2.76	NR	Positive cough stress test and urodynamic confirmation	15	AMDSC	60	12	• Cough test<br>• 1-h pad test<br>• ICIQ-UISF and I-QoL	• Decrease in the mean ICIQ score from 18.06±2.61 to 9.73±6.56<br>• Decrease in the mean I-QoL score from 79.47±17.75 to 46.6±21.89
Garcia-Arranz et al. 2020 [44]	Spain	56.8 ± 9	NR	Clinical history and urodynamic confirmation	10	AASC	50	12	• Cough test<br>• 24-h pad-test<br>• SF-36 and ICIQ-SF<br>• MUCP	• Decrease in pad weight from 22.2 ± 26.7 to 14.3 ± 20<br>• Decrease in MUCP from 44.3 ± 20.3 to 36.4 ± 22.0 cmH2O
Kuismanen et al. 2014 [45]	Finland	59 (45-81)	27 (25-41)	Clinical history, positive cough stress test, and urodynamic testing	5	AASC	90	12	• Cough test with full bladder<br>• 24-h pad-test	• Decrease in pad weight from 22.6 ± 28.3 to 8.8 ± 11.45 g

Arjmand et al. 2017 [46]	Iran	45.8 ± 8.7	27.22 ± 3.8	SUI symptoms for >2 years and positive stress and Q-tip tests; urodynamics performed only in complicated cases	10	AASC	38.6	6	• 24-h pad test<br>• ICIQ-SF	• Significant decrease in mean pad weight from baseline to 2 weeks post-injection, no change at 6 weeks, and a significant increase in at 24 weeks.<br>• Significant decrease in the mean ICIQ scores from baseline at 2 weeks that remained sustained through the 24-week follow-up
Mahboubeh et al. 2023* [47]	Iran	51.93 ± 10.8	NR	SUI symptoms, observed leakage during cough stress testing, and urodynamic testing	15	AOMSC	64	24	• cough stress test with a full bladder<br>• IIQ-7	• Decrease in the mean IIQ-7 score from baseline from 66.73 ± 7.72 to 54.26 ± 17.08 ($p = 0.06$).
Lee et al. 2010 [48]	South Korea	51.5 ± 7.6	NR	Not specified	39	ACBSC	430	12	• Satisfaction Survey (non-validated)<br>• MUCP	• Increase in MUCP compared to baseline from 20.3 ± 5.9 cmH2O to 45.0 ± 9.7 cmH2O at 3 months postoperatively
Souza et al. 2026a [49]	Brazil	56.6 ± 10.2	33.7 ± 8.5	Positive cough stress test and standardized pad testing	11	AMDSC	10	12	• Cough stress test<br>• 20-minute pad test<br>• MUCP	• Decrease in pad weight from 8.3 ± 10.3 to 2.5 ± 5.5 g<br>• Increase in MUCP from 68.6 ± 14.4 to 79.9 ± 19.4 cmH2O
Souza et al. 2026b [49]	Brazil	49 ± 10.8	28.9 ± 6.2	Positive cough stress test and standardized pad testing	9	BMSC	10	12	• Cough stress test<br>• 20-minute pad test<br>• MUCP	• Decrease in pad weight from 10.4 ± 10.9 to 2.4 ± 3.0 g<br>• Increase in MUCP from 63.1 ± 17.4 to 66.9 ± 21.4 cmH2O

Note: Data are presented as mean ± SD, or median (range) as reported in the respective primary studies. Abbreviations: **AMDSC**, autologous muscle-derived stem cells; **AASC**, autologous adipose-derived stem cells, **AOMSC**: autologous oral mucosa derived stem cells, **ACBSC:**

autologous cord blood-derived stem cells; **IEF**, incontinence episode frequency; **MUCP**, maximum urethral closure pressure; **VLPP**, Valsalva leak point pressure; **CLPP**, cough leak point pressure; **UDI-6**, Urogenital Distress Inventory-6; **IIQ-7**, Incontinence Impact Questionnaire-7; **ICIQ-UI-SF**, International Consultation on Incontinence Questionnaire-Urinary Incontinence Short Form; **IQoL / I-QOL**, Incontinence Quality of Life; **NR**, not reported; **BMSC,** bone marrow derived mesenchymal stem cells. Asterisks (*) denote studies with multicenter or comparative trial designs.

Table S2A. Risk of bias assessment of randomized controlled trials using Cochrane RoB 2.

**First author year [Ref]**	**D1**	**D2**	**D3**	**D4**	**D5**	**Overall risk of bias**	**Note**
Kaufman et al.2024 [29]	Low	Low	Low	Low	Low	**Low**	Centralized randomization; double-blind placebo-controlled design; balanced groups; minimal missing 12-month data; prespecified primary endpoint.
Jankowski et al. 2018 [36]	Low	Low	Low	Low	Low	**Low**	Prespecified block randomization with IVRS; double-blind placebo-controlled design; minimal attrition; relevant efficacy endpoint prespecified.
Rose & Rübben 2025 [30]	Low	High	High	High	Low	**High**	Central randomization, but open allocation to cell vs control groups; substantial post-randomization exclusions; participant-reported incontinence episodes in an unblinded comparison.
Daneshpajooh et al. 2022 [43]	Some concerns	Low	Low	Low	Some concerns	**Some concerns**	Random-number allocation, but concealment unclear; outcome assessor and analyst blinded; complete follow-up; prespecified analysis plan unclear.
Mahboubeh et al. 2023 [47]	Low	Low	Low	Low	Some concerns	**Some concerns**	Sequentially numbered allocation prepared by statistician; blinded follow-up assessor; complete follow-up; prespecified analysis plan unclear.

RoB 2 assessments were performed for the five domains: D1, bias arising from the randomization process; D2, bias due to deviations from intended interventions; D3, bias due to missing outcome data; D4, bias in measurement of the outcome; D5, bias in selection of the reported result.

Reference:

Sterne JAC, Savović J, Page MJ, Elbers RG, Blencowe NS, Boutron I, et al. RoB 2: a revised tool for assessing risk of bias in randomised trials. *BMJ*. 2019;366:l4898.

Table S2B. Critical appraisal of prospective uncontrolled studies using the JBI Critical Appraisal Checklist for Case Series.

First author year [Ref]	Q1	Q2	Q3	Q4	Q5	Q6	Q7	Q8	Q9	Q10
Schmid et al. 2023 [33]	**Y**	**Y**	**Y**	**NA**	**Y**	**Y**	**Y**	**Y**	**Y**	**Y**
Blaganje et al. 2022 [32]	**Y**	**Y**	**Y**	**U**	**Y**	**Y**	**Y**	**Y**	**Y**	**Y**
Arjmand et al. 2017 [46]	**Y**	**Y**	**Y**	**U**	**Y**	**Y**	**Y**	**Y**	**Y**	**Y**
Carr et al. 2008 [42]	**Y**	**Y**	**Y**	**U**	**Y**	**Y**	**Y**	**Y**	**Y**	**Y**
Sharifiaghdas et al. 2019 [38]	**Y**	**Y**	**Y**	**U**	**Y**	**Y**	**Y**	**Y**	**Y**	**Y**
Garcia-Arranz et al. 2020 [44]	**Y**	**Y**	**Y**	**U**	**Y**	**Y**	**Y**	**Y**	**Y**	**Y**
Dutta et al. 2025 [35]	**Y**	**Y**	**Y**	**U**	**Y**	**Y**	**Y**	**Y**	**Y**	**Y**
Kuismanen et al. 2014 [45]	**U**	**Y**	**Y**	**N**	**Y**	**Y**	**Y**	**Y**	**Y**	**Y**
Peters et al. 2014 [40]	**Y**	**Y**	**Y**	**U**	**Y**	**Y**	**Y**	**Y**	**Y**	**Y**
Sèbe et al. 2011 [41]	**Y**	**Y**	**Y**	**U**	**Y**	**Y**	**Y**	**Y**	**Y**	**Y**

Stangel-Wójcikiewicz et al. 2014 [37]	**Y**	**Y**	**Y**	**U**	**U**	**Y**	**Y**	**U**	**Y**	**Y**
Sharifiaghdas et al. 2016 [39]	**Y**	**Y**	**Y**	**U**	**Y**	**Y**	**Y**	**Y**	**Y**	**Y**
Carr et al. 2013 [31]	**Y**	**Y**	**Y**	**U**	**Y**	**Y**	**Y**	**Y**	**Y**	**Y**
Lee et al. 2010 [48]	**U**	**Y**	**U**	**U**	**N**	**Y**	**Y**	**U**	**Y**	**U**
Souza et al. 2026 [49]	**Y**	**Y**	**Y**	**U**	**Y**	**Y**	**Y**	**Y**	**Y**	**Y**
Mitterberger et al. 2007 [34]	**Y**	**Y**	**Y**	**U**	**Y**	**Y**	**Y**	**Y**	**Y**	**Y**

JBI checklist items

Item	Question
Q1	Were there clear criteria for inclusion in the case series?
Q2	Was the condition measured in a standard, reliable way for all participants included in the case series?
Q3	Were valid methods used for identification of the condition for all participants included in the case series?
Q4	Did the case series have consecutive inclusion of participants?
Q5	Did the case series have complete inclusion of participants?
Q6	Was there clear reporting of the demographics of the participants in the study?
Q7	Was there clear reporting of clinical information of the participants?
Q8	Were the outcomes or follow-up results of cases clearly reported?
Q9	Was there clear reporting of the presenting site(s)/clinic(s) demographic information?
Q10	Was statistical analysis appropriate?

Key: Y = Yes; N = No; U = Unclear; NA = Not applicable.

Reference:

Munn Z, Barker TH, Moola S, Tufanaru C, Stern C, McArthur A, et al. Methodological quality of case series studies: an introduction to the JBI critical appraisal tool. JBI Evid Synth. 2020;18(10):2127-33.

Table S2C. GRADE summary of findings and certainty of evidence for stem cell–based urethral injection therapy in women with stress urinary incontinence.

Outcome	Design	Risk of bias	Inconsistency	Indirectness	Imprecision	Other	Effect	Certainty
Post-treatment response after cell injection (single-arm proportion)	5 RCTs + 12 uncontrolled prospective studies	Serious1	Serious2	Serious3	Not serious	None	Pooled 63.7% (95% CI 56.7–70.5)	**Very low** ⊕◯◯◯
≥50% reduction in SUI episode frequency at 12 months: cell therapy vs placebo	2 RCTs	Not serious	Not serious	Not serious	Serious4	None	RR 1.02 (0.85–1.23)	**Moderate** ⊕⊕⊕◯
Clinical response: cell therapy vs sling surgery	2 RCTs	Serious5	Not serious	Not serious	Very serious6	None	Not pooled. Overall improvement at 12 months was 66.6% cell vs 93.3% sling in one trial. The other trial reported a negative Marshall test in 73% vs 80%.	**Very low** ⊕◯◯◯
Safety of cell-based injection therapy	5 RCTs + 16 uncontrolled studies	Serious7	Serious8	Not serious	Serious9	None	Events mostly mild and transient. Three serious events, all in Kaufman et al. No treatment-related deaths.	**Very low** ⊕◯◯◯

Footnotes

1. The uncontrolled studies had unclear or non-consecutive recruitment on the JBI checklist. Among the contributing RCTs, Rose & Rübben was at high risk of bias, and Daneshpajooh and Mahboubeh had some concerns.

2. $I^2 = 62.6\%$ ($p < .001$). Cohort response rates ranged from 47% to 79%, and the subgroup differences were confounded, so they don't explain the heterogeneity.
3. A pooled improvement rate does not isolate a treatment-specific effect.
4. Only two trials contributed. The CI is compatible with a 15% relative reduction to a 23% increase, and the placebo response was much higher than anticipated (53.6% vs 30%).
5. Both trials were rated "some concerns".
6. Only 60 participants in total. The trials used different cell products, different slings and different outcomes, so no pooled estimate was possible.
7. Mostly uncontrolled data with non-standardized adverse-event reporting. Some studies reported participants, others episodes, and some only related events.
8. Extreme variations in reported rates for the same adverse events.
9. Most studies were small and not powered for rare events, and follow-up was limited (3–48 months), so delayed complications can't be excluded.

Reference:

Schünemann H, Brożek J, Guyatt G, Oxman A, editors. GRADE handbook for grading quality of evidence and strength of recommendations. Updated October 2013. The GRADE Working Group; 2013. Available from: https://gradepro.org/handbook/

Table S3. PRISMA 2020 Checklist.

Section and Topic	Item #	Checklist item	Location where item is reported
**TITLE**			
Title	1	Identify the report as a systematic review.	Page 3
**ABSTRACT**			
Abstract	2	See the PRISMA 2020 for Abstracts checklist.	Page 1
**INTRODUCTION**			
Rationale	3	Describe the rationale for the review in the context of existing knowledge.	Page 3
Objectives	4	Provide an explicit statement of the objective(s) or question(s) the review addresses.	Page 3
**METHODS**			
Eligibility criteria	5	Specify the inclusion and exclusion criteria for the review and how studies were grouped for the syntheses.	Page 4
Information sources	6	Specify all databases, registers, websites, organisations, reference lists and other sources searched or consulted to identify studies. Specify the date when each source was last searched or consulted.	Page 4
Search strategy	7	Present the full search strategies for all databases, registers and websites, including any filters and limits used.	Page 4
Selection process	8	Specify the methods used to decide whether a study met the inclusion criteria of the review, including how many reviewers screened each record and each report retrieved, whether they worked independently, and if applicable, details of automation tools used in the process.	Page 4
Data collection process	9	Specify the methods used to collect data from reports, including how many reviewers collected data from each report, whether they worked independently, any processes for obtaining or confirming data from study investigators, and if applicable, details of automation tools used in the process.	Page 5
Data items	10a	List and define all outcomes for which data were sought. Specify whether all results that were compatible with each outcome domain in each study were sought (e.g. for all measures, time points, analyses), and if not, the methods used to decide which results to collect.	Page 5
	10b	List and define all other variables for which data were sought (e.g. participant and intervention characteristics, funding sources). Describe any assumptions made about any missing or unclear information.	Page 5
Study risk of bias assessment	11	Specify the methods used to assess risk of bias in the included studies, including details of the tool(s) used, how many reviewers assessed each study and whether they worked independently, and if applicable, details of automation tools used in the process.	Page 5
Effect measures	12	Specify for each outcome the effect measure(s) (e.g. risk ratio, mean difference) used in the synthesis or presentation of results.	Page 5

Section and Topic	Item #	Checklist item	Location where item is reported
Synthesis methods	13a	Describe the processes used to decide which studies were eligible for each synthesis (e.g. tabulating the study intervention characteristics and comparing against the planned groups for each synthesis (item #5)).	Page 5-6
	13b	Describe any methods required to prepare the data for presentation or synthesis, such as handling of missing summary statistics, or data conversions.	Page 5-6
	13c	Describe any methods used to tabulate or visually display results of individual studies and syntheses.	Page 5-6
	13d	Describe any methods used to synthesize results and provide a rationale for the choice(s). If meta-analysis was performed, describe the model(s), method(s) to identify the presence and extent of statistical heterogeneity, and software package(s) used.	Page 5-6
	13e	Describe any methods used to explore possible causes of heterogeneity among study results (e.g. subgroup analysis, meta-regression).	Page 5-6
	13f	Describe any sensitivity analyses conducted to assess robustness of the synthesized results.	Page 5-6
Reporting bias assessment	14	Describe any methods used to assess risk of bias due to missing results in a synthesis (arising from reporting biases).	Page 5
Certainty assessment	15	Describe any methods used to assess certainty (or confidence) in the body of evidence for an outcome.	Page 5
**RESULTS**			
Study selection	16a	Describe the results of the search and selection process, from the number of records identified in the search to the number of studies included in the review, ideally using a flow diagram.	Page 6-7
	16b	Cite studies that might appear to meet the inclusion criteria, but which were excluded, and explain why they were excluded.	Page 6-7
Study characteristics	17	Cite each included study and present its characteristics.	Page 7-8
Risk of bias in studies	18	Present assessments of risk of bias for each included study.	Page 8
Results of individual studies	19	For all outcomes, present, for each study: (a) summary statistics for each group (where appropriate) and (b) an effect estimate and its precision (e.g. confidence/credible interval), ideally using structured tables or plots.	Page 8-12
Results of syntheses	20a	For each synthesis, briefly summarise the characteristics and risk of bias among contributing studies.	Page 8
	20b	Present results of all statistical syntheses conducted. If meta-analysis was done, present for each the summary estimate and its precision (e.g. confidence/credible interval) and measures of statistical heterogeneity. If comparing groups, describe the direction of the effect.	Page 8-12
	20c	Present results of all investigations of possible causes of heterogeneity among study results.	Page 8-9,11-12
	20d	Present results of all sensitivity analyses conducted to assess the robustness of the synthesized results.	Page 12
Reporting biases	21	Present assessments of risk of bias due to missing results (arising from reporting biases) for each synthesis assessed.	Page 8
Certainty of evidence	22	Present assessments of certainty (or confidence) in the body of evidence for each outcome assessed.	Page 8-12, Supplementary

Section and Topic	Item #	Checklist item	Location where item is reported
			Table S2C
**DISCUSSION**			
Discussion	23a	Provide a general interpretation of the results in the context of other evidence.	Page 13-18
	23b	Discuss any limitations of the evidence included in the review.	Page 18
	23c	Discuss any limitations of the review processes used.	Page 19
	23d	Discuss implications of the results for practice, policy, and future research.	Page 19
**OTHER INFORMATION**			
Registration and protocol	24a	Provide registration information for the review, including register name and registration number, or state that the review was not registered.	Page 3
	24b	Indicate where the review protocol can be accessed, or state that a protocol was not prepared.	Page 3
	24c	Describe and explain any amendments to information provided at registration or in the protocol.	Page 3
Support	25	Describe sources of financial or non-financial support for the review, and the role of the funders or sponsors in the review.	Page 20
Competing interests	26	Declare any competing interests of review authors.	Page 20
Availability of data, code and other materials	27	Report which of the following are publicly available and where they can be found: template data collection forms; data extracted from included studies; data used for all analyses; analytic code; any other materials used in the review.	Page 20

From: Page MJ, McKenzie JE, Bossuyt PM, Boutron I, Hoffmann TC, Mulrow CD, et al. The PRISMA 2020 statement: an updated guideline for reporting systematic reviews. BMJ 2021;372:n71. doi: 10.1136/bmj.n71. This work is licensed under CC BY 4.0. To view a copy of this license, visit https://creativecommons.org/licenses/by/4.0/

Figure S1. RoB 2 traffic-light plot.

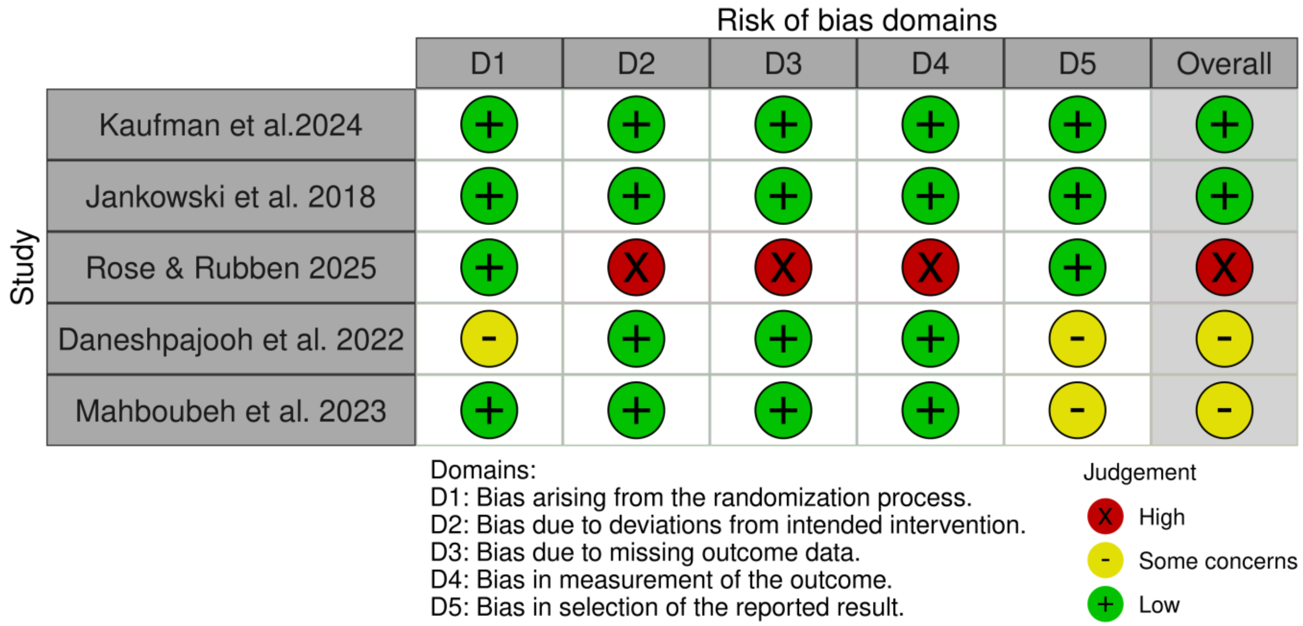

Reference:

McGuinness LA, Higgins JPT. Risk-of-bias VISualization (robvis): An R package and Shiny web app for visualizing risk-of-bias assessments. Res Synth Methods. 2021;12(1):55–61. doi:10.1002/jrsm.1411.
